# General Therapeutics and Materia Medica, Adapted for a Medical Text-Book

**Published:** 1844-01

**Authors:** 


Art. III.—*General Therapeutics and Materia Medica, adapted for a Medical Text-book.* By Robley Dunglison, m.d., Professor of Institutes of Medicine in Jefferson Medical College, Philadelphia, &c. —*Philadelphia*, 1843. Two Vols. 8vo, pp. 988.

The works of Dr. Dunglison are of a very uniform character. They are those of a well-informed, industrious, and judicious professor of medicine, who aptly perceives the kind of books that are wanted within the sphere of his own activity, and who is well qualified to supply the desiderata. We by no means intend to insinuate, however, that Dr. Dunglison's works are not calculated to be everywhere useful. The volumes before us consist of a republication of the author's work on 'General Therapeutics,' with a new treatise on 'Materia Medica.' Only seven years having elapsed since the first appearance of the former, little addition or alteration has been required; for changes in our views of general therapeutics come round but slowly, while every month brings forth some novelty in the specialities of the healing art. The subject of materia medica has been handled by our author with more than usual judgment. The greater part of treatises on that subject are, in effect, expositions of the natural and chemical history of the substances used in medicine, with very brief notices of their medicinal virtues, and scarcely any notice at all of the relations which obtain between the special indications they are capable of fulfilling, and the general principles of therapeutics. In short, such books are adapted merely for the apothecary. Dr. Dunglison, very wisely in our opinion, has reversed all this, and given his principal attention to the articles of the materia medica as *medicines*. Nevertheless he has, in all instances, "referred to the position held by the drug as an article of the organized or of the inorganic kingdom, as well as to general matters of interest relative to the place where it is found; the manner in which it is obtained; and to certain points connected with its commercial history; but next to therapeutical applications, he has dwelt more at length on the sensible properties by which the physician may be enabled to judge of the various articles from his own observation." (Preface.)

Dr. Dunglison has succeeded very happily in connecting general with special therapeutics. His work is cast in a somewhat similar mould with that of Dr. A. T. Thomson on the same subjects. If the latter have, perhaps, the advantage in plenitude of materials, the former is more lucid in its arrangement and simpler in its expositions; but as the materia medica of the one is conformed to the British Pharmacopœias, and of the other to that of the United States, there is ample room for them both. Dr. Dunglison has modified the classification of therapeutical agents, adopted in his 'General Therapeutics,' when first published. It now stands as follows:

| | |
|---|---|
| 1. Agents that affect prominently the alimentary canal or its contents | Emetics.<br>Cathartics.<br>Anthelmintics. |
| 2. Agents that affect prominently the respiratory organs . | Expectorants. |
| 3. Agents that affect prominently the follicular or glandular organs | Errhines.<br>Sialogogues.<br>Diuretics.<br>Antilithics.<br>Diaphoretics. |
| 4. Agents that affect prominently the nervous system . | Narcotics.<br>Tetanics.<br>Antispasmodics. |
| 5. Agents that affect prominently the organs of reproduction . | Emmenagogues.<br>Parturients. |
| 6. Agents that affect various organs . . . | Excitants.<br>Tonics.<br>Astringents.<br>Sedatives.<br>Refrigerants.<br>Revellents.<br>Eutrophics. |
| 7. Agents whose action is prominently chemical . . | Antacids.<br>Antalkalies.<br>Disinfectants. |
| 8. Agents whose action is prominently mechanical . . | Demulcents.<br>Diluents. |

We do not attach much importance to arrangements of this nature, which, in the present state of our knowledge, cannot even approximate to accuracy. All successful attempts at classification must be based on the recognition of certain distinctive and invariable characters in the objects classified: how little this is the case with respect to therapeutic agents, it would be superfluous to demonstrate. Dr. Dunglison has here introduced two new terms. To one of these we decidedly object, namely, *parturients*, the use of which would imply that the therapeutic agents themselves were about to bring forth infant remedies. It would not be easy to find an appropriate name for such agents, for the Greek language, as far as we know, contains no verb signifying "to cause to bring forth," nor does it afford any combination which would aptly designate the agents in question, unless, indeed, it be *tocogogues*, from *τοκος partus*, and *αγω duco;* we believe, after all, that worse terms have passed current. The other term to which we allude is *eutrophics*, (promoters of healthy nutrition,) which Dr. Dunglison has substituted, judiciously we think, for "alteratives," a term conveying no distinct meaning.

In conclusion, we strongly recommend these volumes to our readers. No medical student, on either side of the Atlantic, ought to be without them.

